# Pulp Temperature Rise Induced by Light-Emitting Diode Light-Curing Units Using an Ex Vivo Model

**DOI:** 10.3390/ma12030411

**Published:** 2019-01-29

**Authors:** Alexandra Vinagre, João C. Ramos, Clara Rebelo, José Francisco Basto, Ana Messias, Nélia Alberto, Rogério Nogueira

**Affiliations:** 1 Dentistry Area, Faculty of Medicine, University of Coimbra, Avenida Bissaya Barreto, Blocos de Celas, 3000-075 Coimbra, Portugal; joaoctramos@sapo.pt (J.C.R.); clararebelo.md@gmail.com (C.R.); ana.messias@uc.pt (A.M.); 2 IPMD, Instituto Português de Medicina Dentária, Rua José Luciano Castro, nº 141, Esgueira, 3800-207 Aveiro, Portugal; franciscobasto.md@gmail.com; 3 Instituto de Telecomunicações, Campus Universitário de Santiago, P-3810-193 Aveiro, Portugal; nelia@ua.pt (N.A.); rnogueira@av.it.pt (R.N.)

**Keywords:** dental pulp, LED light-curing unit, light curing, temperature rise, optical fiber sensors, fiber Bragg gratings

## Abstract

The aim of this research was to compare the pulp temperature (PT) rise induced by four light-emitting diode light-curing units (LED LCUs) (Bluephase 20i, Demi Ultra, SPEC 3, and Valo) in different curing modes. Immediately after extraction, the pulp chamber of 11 premolars was accessed from the palatal cervical third of the crown for insertion of fiber Bragg grating (FBG) sensors for temperature measurement and kept in a 37.0° water bath. The teeth were then submitted to a random sequence of curing modes with four irradiations at 30 s intervals. Care was taken to ensure complete pulp temperature reset between curing modes. The curing modes were classified as high-energy (above 80 J/cm^2^) or low-energy (below 40 J/cm^2^) according to the total energy density delivered. Statistical analysis was performed with repeated ANOVA measures and Pearson’s correlation for the association between energy density and temperature variation. The significance level was set to 0.05. All curing units promoted a statistically significant PT rise (p < 0.01). After four emissions, the PT rise was higher than 5.0 °C for the high-energy curing modes. The low-energy modes induced approximately a 2.5 °C rise. A strong positive correlation was found between energy density and PT increase (R = 0.715; p = 0.01). Exposure of intact premolars to LED LCUs induced significant and cumulative PT rise. Curing modes emitting high energy densities produced higher PT variations. Radiant exposure was positively correlated to PT variation.

## 1. Introduction

A large number of current daily clinical procedures can cause pulp temperature (PT) rise, with subsequent development of symptoms like hyperalgesia, dentinal hypersensitivity, and spontaneous pain typical of acute pulpitis due to pulp heating and trauma [1,2]. Burn reactions at the periphery of the pulp can include injuries on the odontoblastic layer leading to degeneration, protoplasm coagulation, expansion of liquid in the dentinal tubules, and pulp with increased outward flow from tubules [3].

Even though the in vivo behavior of dentine pulp complex in response to a thermal stimulus depends on several factors, such as the remaining dental thickness, the tooth type, the fluid motion in dentinal tubules, and the pulp microcirculation, PT rise and its effects are highly dependent on the type, intensity, and duration of the applied stimulus [4,5,6,7,8]. For instance, in 1965, Zach and Cohen [9] demonstrated in an animal model that a temperature rise of 5.5 °C in healthy pulps induced necrosis in about 15% of teeth. When the intra-pulpal temperature increase was sustained at 11.1 °C for 10 s, approximately 60 to 70% of teeth developed irreversible pulpitis.

Tooth exposure to light-curing units (LCUs) is one of the routine conditions in restorative dentistry most highly associated with PT increase, which can widely vary from 1.5 °C to 23.2 °C, probably depending on the differential experimental settings in different studies [7,10]. In addition to the heat produced by the LCU, the polymerization of the composite induces an exothermic reaction that has been discussed as a cause of tissue damage. Several studies have reported that the exothermic reaction of the resin-based materials during polymerization heightens as the amount of filler present in the resins diminishes. Additionally, a proportional relationship has been established between the temperature rise from the exothermic reaction and the amount of composite resin applied. Therefore, the possibility of pulpal damage caused by temperature rise should be considered when using light-cured bulk-fill materials in very deep cavities. In this clinical situation, it is suggested to avoid placing a large amount of composite as a first layer [11,12,13]. Notwithstanding, composite resin increments added to a restoration act as insulators rather than heat generators from an exothermic reaction. This is corroborated by other studies that indicate that the heat generated from the LCU has much more impact than the heat from the exothermic reaction of the composite resin itself. The energy density delivered by the LCU plays the most important role in intra-pulpal temperature rise [5,14,15,16].

The convenience of light-emitting diode (LED) LCUs makes them increasingly popular for curing dental restorative materials. Currently, second and third generation LED units have progressively replaced older incandescent halogen lamps because of their inherent advantages, namely, energy efficiency, long life span, and ergonomics. LED technology can also incorporate high-intensity chip sets placed either in the body or at the distal tip of a gun or pen-type device [17]. Though no infrared energy is present in the light emitted by these devices, a considerable amount of heat may still be expected at the target, since they can reach power densities above 2000 mW/cm^2^ [10]. Despite the differences in light output between quartz–tungsten–halogen (QTH) and LED curing units, variations in PT rise have been linked more to higher radiant exitances and exposure times than to the light beam profile itself [5,8,16].

Several methods have been applied for measuring the temperature rise in pulp tissues, such as thermocouples, differential thermal analysis, differential scanning calorimetry, and infrared thermal camera [4,7,18]. Optical fiber Bragg grating (FBG)-based sensors can be used to measure a wide variety of physical quantities (e.g., strain, temperature, vibration, pressure, acceleration, and refractive index) [19,20] and offer good performance under extreme conditions thanks to their small dimensions, low weight, biocompatibility, resolution, immunity to electromagnetic interference, chemical inertia, and spark-free properties [21].

This study aimed to compare the pulp chamber temperature rise induced by LED LCUs in different curing modes using FBG sensors. The tested null hypotheses were: (1) all curing modes of the LED LCUs do not induce a significant PT rise in relation to the baseline temperature; (2) the different curing protocols tested do not produce statistically significant differences in PT variation; (3) there is no relation between delivered radiant energy and PT increase.

## 2. Results

**Figure 1:**
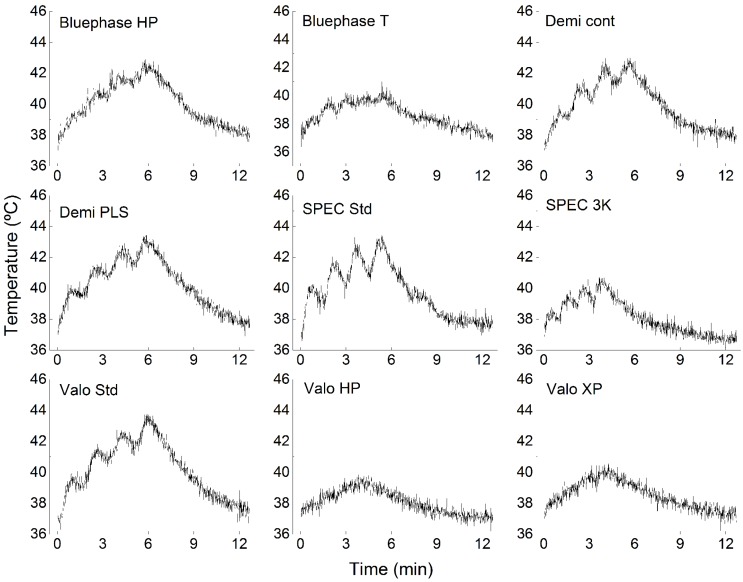
Representative real-time graphic profiles of pulp temperature (PT) increase during the curing protocol for all exposure modes.

The mean values and standard deviations of PT at the end of each light emission (T1, T2, T3, T4) are presented in Table 1 for high-energy-level modes and Table 2 for low-energy-level modes. Representative real-time profiles of PT rise for a representative sample under the different curing modes can be observed in Figure 1.

**Figure 2:**
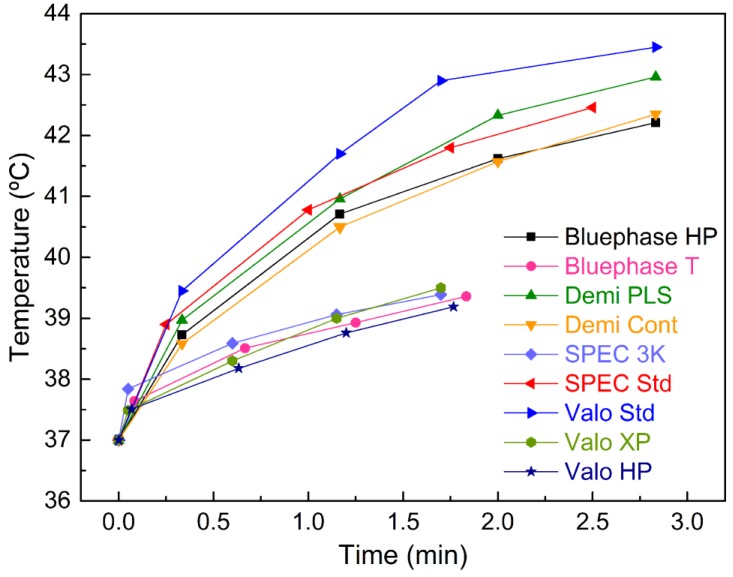
Mean pulp temperature values after each period of light exposure for all LED LCUs and exposure modes.

For the high-energy modes, there was a significant temperature rise from T1 to T4 (F(1.55, 77.33) = 621.27, p < 0.01), with a similar pattern for all, as depicted by the non-significant interaction term (F(6.19, 77.33) = 0.88, p = 0.52) and graphical representation (Figure 2).

Similarly, there was a significant PT rise for the low-energy modes (F(1.90, 76.07) = 211.02, p < 0.01) with no interaction (F(5.70, 76.07) = 0.89, p = 0.50) (Figure 2).

At each evaluation period, no statistically significant differences were detected between curing modes in both the high- and the low-energy-level subsets (p value specified in the bottom row of Table 1 and Table 2).

Energy density was determined for every evaluation time (T1 to T4) of the different curing modes of the four LCUs and associated with the corresponding temperature variation using Pearson’s Correlation. A strong positive correlation was established between these two variables (R = 0.715), which was statistically significant at the p = 0.01 level.

## 3. Discussion

High-irradiance modes have been developed in LED LCU devices, raising concerns about the potential side effects in pulp tissue. The thermal changes during light curing are recognized, but there is a lack of information about temperature changes in pulp tissue under different curing protocols and applications. In the present study, pulp temperature variation induced by the use of four different light units was measured. All tested LED LCU curing modes induced a significant PT rise in relation to the baseline temperature. Therefore, the first null hypothesis was rejected. However, the variation associated with the exposure modes delivering low energy levels (Bluephase T, SPEC 3K, Valo HP, and Valo XP) was not higher than 2.5 °C after four emissions, which might be considered clinically insignificant. On the contrary, all other high-energy-level curing modes produced PT variations around or above 5.5 °C at the same evaluation period and must be viewed as critical or potentially causing pulp damage [9].

Electromagnetic radiation emitted by LCUs should be well understood by professionals, as it can comprise multiple settings with distinct outputs within different spectral emissions. Further, light output and beam profiles can fluctuate significantly during an exposure cycle. Ideally, laboratory-grade devices should be used to characterize real-time spectral radiant power and spectral emissions of LCUs [22,23]. Nevertheless, this equipment is not available in regular dental offices, and manufacturers should be encouraged to provide more accurate information about the light in all available settings. Hand-held radiometers can be used to monitor changes in the light output of LCUs over time, but care should be taken in the validation of the absolute irradiance value. Shimokowa et al. reported a significant variation between the irradiance values provided by radiometers when compared to a laboratory-grade meter, except for the Bluephase Meter II, which provided accurate data [23]. For that reason, the Bluephase Meter II was chosen for irradiance measurement in the present study.

Most contemporary LCUs can operate in either continuous or modulated curing modes, such as the step, ramp, pulse, or pulse-delay approaches. The latter were introduced in an attempt to reduce polymerization shrinkage stress by delivering light exposure at a reduced light output level, extending the pre-gel phase. In addition, they can potentially reduce adverse heating effects [22,24,25]. In the present study, only Demi Ultra was tested in a pulse light mode (periodic level shifting—PLS), slowing the rate of energy by alternating output between low (1100 mW/cm^2^, manufacturer’s indication) and high (1330 mW/cm^2^, manufacturer’s indication) levels of irradiance. Nevertheless, the radiometer used was not able to differentiate between the two levels of irradiance. Accordingly, this mode presented similar results to those obtained with the high-level group. No advantages could be detected in the prevention of PT rise, probably because of the high baseline irradiance level.

Direct comparison of the present results with those reported in the literature is difficult because of the different methodologies employed for temperature measurement and the different tooth preparations. Most studies with similar purposes mainly resort to thermocouples to assess pulp temperature variations. This technique requires removal of the pulp tissues and the use of a conductive material to attach the thermocouple to the dentinal wall, which indirectly assesses pulp temperature as the temperature of the surrounding dentin at frequencies between 0.5 and 1 Hz [4,5,8,18,26,27,28]. On the contrary, the FBG sensors used in the present study can be placed in direct contact with the pulp tissues, allowing for real-time monitoring of the temperature variations at a 2 Hz frequency. Our results are in line with the in vivo study of Runnacles et al., where real-time measurement of thermal variations, by the application of a probe directly into the tissue of the pulp chamber of intact premolars, was also performed. Runnacles et al. showed an average temperature variation of 4.8 °C after a 60 s exposure in the high-power mode (74.64 J/cm^2^) with a single LED LCU (Bluephase 20i) [7].

It is also important to note that during the 30 s rest periods between light emissions, pulp temperature did not recover to baseline levels. This has also been observed in other studies [7,18]. Instead, PT continued to increase until a plateau was reached about halfway through each rest period. Afterwards, PT decreased approximately 1 °C until a new light emission started. On one hand, this effect could be a consequence of the insulating characteristics of enamel and dentin (which have low thermal conductivity and diffusivity), leading to the retention of thermal energy transferred by the light emission [29] in teeth without cavity preparation. On the other hand, this effect could have been caused by the absence of blood microcirculation within pulp tissues. Though the premolars used were tested within a very short period after the extraction, blood supply to the pulp was interrupted. This led to stasis in the pulp chamber and diminished the cooling capacity to dissipate the heat transferred by external thermal stimuli, as identified in several studies reporting experimental models of pulpal microcirculation [4,6]. Clinically, it is possible that healthy teeth with proper blood microcirculation present better cooling conditions, as they do not undergo high temperature variations when exposed to LCUs and recover faster to baseline levels. However, in direct restorative procedures, the remaining dentin and enamel barrier between the light source and the pulp is thinner. In association with the additional heat produced by the exothermic reaction of resin-composite polymerization, this might contribute to higher temperature variations in the pulp and potential damage [16,30,31]. In this case, larger cavities are associated with higher heat generated by the application of resin-composite increments and sustained light energy delivered [32]. This reflects the direct relationship between radiant exposure (J/cm^2^), which is the product of light intensity and exposure time, and pulp temperature variation previously described by Runnacles et al. [7]. This relationship was also verified in the present work with a strong positive correlation between the two variables (R = 0.715). Clinicians must be aware that delivering high radiant exposure (above 80 J/cm^2^) to teeth, disregarding recovery periods, might induce PT rise above the acceptable threshold of 5.5 °C. Though it is not expected that pulp temperature rise induced by the irradiance of a tooth causes irreversible pulpitis or necrosis by itself, other factors related to the restorative procedure(e.g., cavity dimensions, adhesive system employed, and filling technique, etc.), could determine the short- and long-term success.

## 4. Materials and Methods

### 4.1. Fiber Bragg Grating (FBG) Sensors

This ex vivo study used FBGs to measure intra-pulpal temperature variation induced by light curing with four LED LCUs.

The FBG consisted of a periodic modulation of the refractive index along the fiber core. Under broadband illumination the FBG reflected the wavelengths that satisfied the Bragg condition and transmitted all others.

The Bragg condition is given by Equation (1):

λ_B_ = 2Λn*_eff_*, (1)

where λ_B_ is the reflected Bragg wavelength, Λ is the periodic modulation of the refractive index, and n*_eff_* is the effective refractive index of the fiber core [33].

Any external perturbation altered the periodicity of the Bragg grating and/or the effective refractive index of the fiber core, changing the reflected Bragg wavelength. Using the first equation, the shift in the Bragg wavelength due to strain and temperature changes is given by Equation (2):

(2) $$\Delta \;\lambda_{B}=\Delta \;\lambda_{B,l}+\Delta \;\lambda_{B,T}=2\left(\Lambda \frac{\partial n}{\partial l}+n\frac{\partial \Lambda }{\partial l}\right)\Delta l+2\left(\Lambda \frac{\partial n}{\partial T}+n\frac{\partial \Lambda }{\partial T}\right)\Delta T=S_{l}\Delta l+S_{T}\Delta T,$$

where Δ*λ_B,l_* and Δ*λ_B,T_* are the strain- and temperature-induced wavelength shifts, respectively. The shift of the Bragg wavelength due to strain was mainly associated with changes in the grating period, whereas the wavelength shift due to temperature variation was mainly justified by the corresponding shift in the refractive index. $S_{l}$ and $S_{T}$ are constant values and represent the strain and temperature sensitivity coefficients of the FBG sensors.

In the current study, FBGs with 1 mm length were inscribed onto photosensitive optical fiber (FiberCore PS 1250/1500, Southampton, United Kingdom) with a UV light (248 nm) from a KrF excimer laser (BraggStar Industrial, LN, Santa Clara, USA), using the phase mask grating inscription method. The optical fiber consisted of a cylindrical structure with two layers—the core and cladding, with 9.6 and 125.0 μm of diameter, respectively. To improve the mechanical resistance, the optical fiber was coated with a polymer, bringing the overall diameter to 250.0 μm. This polymer was previously removed in the FBG region before the inscription process.

Since no strain was induced in the FBG sensor, the entire Bragg wavelength variation was only temperature-driven. Consequently, the first term of Equation (2) was considered null. The FBGs were thermally characterized using a climatic chamber (CH340, Angelantoni Industrie, Massa Martana, Italy), aiming to determine the temperature sensitivity coefficient. The temperature was increased from 15.0 to 60.0 °C with step increments of 5.0 °C. For each temperature level, after a stabilization period of 15 min, the reflected Bragg wavelength was registered. An average temperature sensitivity coefficient of 0.0089 ± 0.0001 nm/°C was obtained.

### 4.2. Specimen Preparation and Temperature Measurement

Eleven freshly extracted intact human maxillary premolars, requiring extraction for orthodontic reasons, were selected for the present study after patient informed consent, as approved by the Ethical Committee of the Faculty of Medicine of Coimbra, Portugal (CE-001/2013). Immediately after the extraction procedure, all surrounding soft tissues were cleaned from the teeth. The pulp chambers were accessed from the cervical third of the palatal aspect of the teeth so that a 25-gauge, 16 mm-long needle (B. Braun Medical Inc, Melsungen, Germany) could be inserted until reaching contact with the buccal circumpulpal dentine inside the pulp chamber. Needle positioning was confirmed with digital radiographs. Afterwards, the tooth was fixated with an individualized support, immersed up to the cemento–enamel junction in a 37.0 ± 0.2 °C distilled thermostatic water bath (Thermostatic bath, model n° 601/3, Nahita, Navarra, Spain), and monitored using an additional digital thermometer (Digital Thermo Sensor Thermometer, Hagen, Montreal, Canada). The FBG sensor was inserted into the pulp chamber through the needle. The needle was then removed, allowing the FBG sensor to stay within the pulp tissue at a 1 mm distance from the chamber wall. The Bragg sensor was connected to an optical sensing interrogator (sm 125-500, MicronOptics Inc., Atlanta, USA), which allowed acquiring, in real time, the Bragg wavelength with a resolution of 1 pm, at a frequency of 2 Hz.

**Figure 3:**
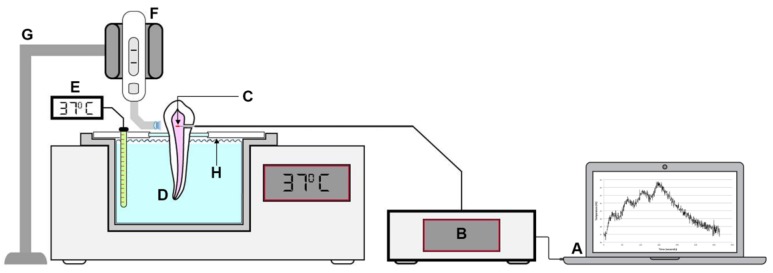
Schematic representation of the setup for PT measurement: **A**—Laptop; **B**—optical sensing interrogator; **C**—FBG sensor; **D**—thermostatic water bath; **E**—digital thermometer for double monitoring; **F**—LED LCU; **G**—LED LCU holder; **H**—individualized tooth support.

The LCUs were placed in a support with the light guide touching the buccal surface of the teeth. The four LED LCUs had a minimum irradiance of 1000 mW/cm^2^ and were used according to the recommendations of the manufacturers in distinct curing modes (Table 3). Per curing mode, four light emissions were made with 30 s intervals. Within each curing protocol, temperature variation was recorded as the increase from 37.0 °C immediately after each light emission period, at the moments designated as T1, T2, T3, and T4. For each mode, emitted irradiance (mW/cm^2^) was measured using a digital radiometer (Bluephase^®^ Meter II, Ser No 130000030, Ivoclar Vivadent, Schann, Liechtenstein), on the basis of the tip diameter of each LCU. The LCUs and corresponding curing modes were applied in random order, resulting in a total of nine modes per tooth. The total energy density delivered was calculated by multiplying the mean power density and the total exposure time. According to these values, the curing modes were grouped in two main frames: high energy level (above 80 J/cm^2^) and low energy level (below 40 J/cm^2^). Care was taken to ensure complete pulp temperature reset between curing protocols. The experimental setup is represented in Figure 3. The room temperature was set to 22.0 °C.

### 4.3. Statistical Analysis

Statistical analysis was performed using IBM^®^ SPSS^®^ Statistics Version 23.0. Differences between homologous peaks of different curing modes were analyzed using the Kruskal–Wallis test followed by all pairwise comparisons. Repeated ANOVA measures with a Greenhouse–Geisser correction was used to determine the effect of exposure time in the variation of temperature and possible interactions with the curing modes, while considering the Bonferroni correction for pairwise comparisons. Pearson’s Correlation was used to establish an association between energy density and temperature variation. The significance level was set to α = 0.05.

## 5. Conclusions

A significant PT rise was detected for all exposure modes when intact premolars were exposed to LED LCUs. Curing modes emitting high energy densities produced PT variations around or above 5.5 °C and might be considered clinically relevant. A positive correlation was found between radiant exposure and pulp temperature variation.

## Figures and Tables

**Table 1:** Mean pulp temperature variation above baseline value measured at the end of each four light-curing steps among high-energy-level curing modes.

Energy Level	LED LCU/Curing Mode	Pulp Temperature Variations (°C)				p
		T1	T2	T3	T4	
**HIGH**	Bluephase HP	1.73 ± 1.44	3.71 ± 1.64	4.62 ± 1.85	5.21 ± 1.73	<0.01
	Demi PLS	1.97 ± 1.53	3.96 ± 1.89	5.33 ± 2.00	5.96 ± 2.32	<0.01
	Demi Cont	1.58 ± 1.46	3.50 ± 1.76	4.87 ± 1.84	5.35 ± 2.29	<0.01
	SPEC Std	1.90 ± 1.46	3.78 ± 1.56	4.80 ± 1.96	5.46 ± 1.96	<0.01
	Valo Std	2.45 ± 1.83	4.70 ± 2.35	5.89 ± 2.51	6.45 ± 2.61	<0.01
	p	0.79	0.65	0.49	0.77	

Curing protocol (four light emissions with an interlude of 30 s between them): T1, end of the first light emission, T2, end of the second light emission, T3, end of the third light emission, and T4, end of the fourth light emission.

**Table 2:** Mean pulp temperature variation above baseline value measured at the end of each four light-curing steps among low-energy-level curing modes.

Energy Level	LED LCU /Curing Mode	Pulp Temperature Variations (°C)				p
		T1	T2	T3	T4	
**LOW**	Bluephase T	0.66 ± 0.56	1.51 ± 0.90	1.93± 0.98	2.36 ± 1.08	<0.01
	SPEC 3K	0.84 ± 0.59	1.59 ±0.78	2.06 ±0.84	2.39 ± 0.75	<0.01
	Valo HP	0.51 ±0.70	1.18 ± 0.81	1.76 ± 0.79	2.19 ± 0.89	<0.01
	Valo XP	0.49 ± 0.97	1.30 ± 0.84	2.00 ± 0.78	2.50 ± 0.95	<0.01
	p	0.49	0.62	0.70	0.85	

Curing protocol (four light emissions with an interlude of 30 s between them): T1, end of the first light emission, T2, end of the second light emission, T3, end of the third light emission, and T4, end of the fourth light emission.

**Table 3:** LED light curing unit characterization: wavelength range, curing modes and protocol, estimated irradiance, and delivered energy density.

Curing Unit & External Tip Diameter	Wavelength (nm)	Curing Mode	Power Density (mW/cm^2^) [Manufacturer’s Value]	Curing Protocol	Energy Density (mJ/cm^2^)
Bluephase 20i 8 mm Ivoclar Vivadent, Schaan, Liechtenstein Batch number #506475	385–515	High Power (Bluephase HP)	1080 [1200]	20 s × 4 (30 s interval)	86,400
		Turbo (Bluephase T)	1790 [2000]	5 s × 4 (30 s interval)	35,800
Demi Ultra 8 mm Kerr, Orange, CA, U.S.A. Batch number #784000644	450–470	Continuous (Demi Cont)	1300 [1215]	20 s × 4 (30 s interval)	104,000
		Periodic Level Shifting (PLS) (Demi PLS)	1350 [1100/1330 oscillation per second]	20 s × 4 (30 s interval)	108,000
SPEC 3 8 mm Coltène Whaledent, Cuyahoga Falls, OH, U.S.A. Batch number #130620166	455–465	Standard (SPEC Std)	1360 [1600]	15 s × 4 (30 s interval)	81,600
		3K (SPEC 3K)	2420 [3000]	3 s × 4 (30 s interval)	29,040
Valo 10 mm Ultradent, South Jordan, UT, U.S.A Batch number # V04639	395–480	Standard (Valo Std)	1130 [1000]	20 s × 4 (30 s interval)	90,400
		High Power (Valo HP)	1610 [1400]	4 s × 4 (30 s interval)	25,760
		Xtra Power (Valo XP)	2710 [3200]	3 s × 4 (30 s interval)	32,520
